# What is important to service users and staff when implementing suicide-focused psychological therapies for people with psychosis into mental health services?

**DOI:** 10.3389/fpsyt.2023.1154092

**Published:** 2023-05-12

**Authors:** Sarah Peters, Yvonne Awenat, Patricia A. Gooding, Kamelia Harris, Leanne Cook, Charlotte Huggett, Steven Jones, Fiona Lobban, Daniel Pratt, Gillian Haddock

**Affiliations:** ^1^Division of Psychology and Mental Health, Faculty of Biology, Medicine and Health, School of Health Sciences, University of Manchester, Manchester, United Kingdom; ^2^Manchester Academic Health Science Centre (MAHSC), Manchester, United Kingdom; ^3^Manchester Centre for Health Psychology, Faculty of Biological, Medical and Health Sciences, University of Manchester, Manchester, United Kingdom; ^4^Greater Manchester Mental Health NHS Foundation Trust, Manchester, United Kingdom; ^5^Lancashire and South Cumbria NHS Foundation Trust, Lancashire, United Kingdom; ^6^Department of Health Research, Lancaster University, Lancaster, United Kingdom

**Keywords:** implementation, talking therapy, psychological therapy, suicidality, suicidal thoughts, suicidal behaviors, psychosis, schizophrenia

## Abstract

**Introduction:**

Suicide is a leading cause of death globally. People with psychosis are at increased risk of suicide death and up to half experience suicidal thoughts and/or engage in suicidal behaviors in their lifetime. Talking therapies can be effective in alleviating suicidal experiences. However, research is yet to be translated into practice, demonstrating a gap in service provision. The barriers and facilitators in therapy implementation require a thorough investigation including the perspectives of different stakeholders such as service users and mental health professionals. This study aimed to investigate stakeholders’ (health professionals and service users) perspectives of implementing a suicide-focused psychological therapy for people experiencing psychosis in mental health services.

**Methods:**

Face-to-face, semi-structured interviews with 20 healthcare professionals and 18 service users were conducted. Interviews were audio recorded and transcribed verbatim. Data were analyzed and managed using reflexive thematic analysis and NVivo software.

**Results:**

For suicide-focused therapy to be successfully implemented in services for people with psychosis, there are four key aspects that need to be considered: (i) Creating safe spaces to be understood; (ii) Gaining a voice; (iii) Accessing therapy at the right time; and (iv) Ensuring a straightforward pathway to accessing therapy.

**Discussion:**

Whilst all stakeholders viewed a suicide-focused therapy as valuable for people experiencing psychosis, they also recognize that enabling successful implementation of such interventions will require additional training, flexibility, and resources to existing services.

## 1. Introduction

There is one death by suicide every 40 s (1). In England and Wales in 2021, there were 5,583 suicide deaths and these were significantly higher than the suicide deaths registered in 2020 (2). Suicide fatalities are elevated in individuals experiencing psychosis (3, 4) and up to 50% of people with psychosis will also have suicidal thoughts and/or behaviors within their lifespan (5, 6). Psychological therapy for psychosis has been endorsed by the UK’s National Institute for Health and Care Excellence (7). Similarly, Cognitive Behavioral Therapy (CBT) specifically for suicidal thoughts and behaviors has been recommended by NICE in the UK (8). In the USA, Dialectical Behavior Therapy (DBT), Cognitive Therapy for Suicide Prevention, and Brief Cognitive Behavioral Therapy have been recommended for people who experience suicidality (9). A National Suicide Prevention Strategies report by the World Health Organization highlighted the need for improved access to evidence-based therapies, such as counseling, DBT, and CBT (10).

The evidence-base for CBT with a specific focus on suicidal experiences (e.g., Cognitive Behavioral Suicide Prevention therapy; CBSP) across numerous clinical settings has been growing. Despite NICE recommendations and developments in evidence supporting particular therapies, they have been sparsely implemented with people with psychosis in UK mental health services and have yet to be translated into routine practice. Taken together, this demonstrates a persistent gap in National Health Service (NHS) mental health care provision. A key step in identifying feasibility of implementing a new treatment or service into routine care is to understand the perspectives of those who deliver, commission, and receive such an intervention (11, 12). Consequently, the aim of the current study was to investigate stakeholders’ views on the perceived potential barriers and facilitators to implementing suicide-focused interventions for people experiencing psychosis.

## 2. Materials and methods

Qualitative, face-to-face, semi-structured interviews were conducted with 38 stakeholders (20 healthcare professionals and 18 service users). Participants were recruited from a multi-site randomized controlled trial investigating a novel suicide-focused psychological therapy for people who experience psychosis [i.e., Cognitive AppRoaches to coMbatting Suicidality (CARMS) (13)]. This afforded the opportunity to investigate the experiences of stakeholders around a novel suicide-focused therapy. The authors assert that all procedures contributing to this work comply with the ethical standards of the relevant national and institutional committees on human experimentation and with the Helsinki Declaration of 1975, as revised in 2008. All procedures involving human patients were approved by the Northwest–Greater Manchester Research Ethics Committee (17/NW/0089). Written, informed consent was obtained from all participants.

### 2.1. Participants

#### 2.1.1. Healthcare professionals

Individuals working across five National Health Service (NHS) trusts delivering mental health care in Northern England, third sector organizations and related services were approached to take part in individual telephone or face-to-face interviews. These sites were also taking part in the CARMS randomized controlled trial of cognitive-behavioral suicide prevention therapy (CBSP) which recruited 292 participants (13). Recruitment for the qualitative was purposive, seeking to include a maximum variance sample representing a range of experiences involved in delivering, managing, referring to, and commissioning psychological services for people with psychosis. We sought individuals from numerous mental health care settings (e.g., in-patient, community, early intervention, and third sector) and disciplines (e.g., nursing, psychiatry, social work, general practice, and clinical psychology). Consenting participants took part in an interview at a convenient time and place.

#### 2.1.2. Service users

Service users with experiences of psychosis and suicidal thoughts and/or behaviors were recruited from community mental health and psychiatric inpatient settings across the Northwest of England as part of the CARMS trial. The inclusion criteria were (i) ICD-10 diagnosis relating to non-affective psychosis, e.g., schizophrenia, schizoaffective disorder; (ii) suicidal thoughts and/or behaviors experienced in the 3 months prior to recruitment; (iii) being under the care of a mental health clinical team (e.g., care co-ordinator and psychiatrist); (iv) aged 18 or older; (v) English speaking; and (vi) able to provide informed consent. Exclusion criteria were (i) dementia or other organic brain disorder; and (ii) current participation in another, similar, clinical trial.

Potential service user participants were invited to take part in the current qualitative study following recruitment to the clinical trial. Of the 18 participants recruited, four were randomized to therapy and 14 to treatment as usual (22 and 78%, respectively). Those randomized to therapy had only had the initial therapy session prior to the interview. Sampling sought to include individuals who had a range of experiences of mental health services, gender, ethnicity, and age. Participants provided self-report data of suicide plans, attempts and thoughts in the past 6 months, and substance use in the past 3-months. Those who took part in an interview was given £10 as a token for their participation.

### 2.2. Data collection

Interviews were conducted by LC and YA, between March 2017 and May 2020, and followed flexible topic guides which were developed in collaboration with an Experts by Experience group comprising individuals with experiences of psychosis and suicidality. Health professional and service user topic guides covered the same topics. Interviews explored their views (and where relevant, experiences) of delivering/receiving suicide-focused psychological talking therapy for people experiencing psychosis, including its perceived utility, accessibility, and perceived implementation barriers. Interviews were audio recorded and transcribed verbatim by a professional transcription company and checked for accuracy by the interviewer and a second researcher (RD). Identifying information was removed from transcripts. Audio recordings were deleted once the data analysis was finalized. The median interview length was 46 min (*range*: 21 min–1 h 17 min).

### 2.3. Analysis

Data collection continued alongside analysis and a constant comparison technique (14) was used whereby new data were compared to existing data and analysis was adjusted accordingly. Data were analyzed by LC, YA, SP, and KH using thematic analysis (15, 16). Transcripts were read and re-read numerous times to achieve data familiarization. Healthcare professionals and service user interview transcripts were coded separately line by line. An initial coding framework was developed from service user data corpus and amended following subsequent healthcare professional interviews to ensure it captured concepts present across the two datasets. This process was repeated using computer NVivo software to organize the analysis. LC and KH examined the interview transcripts again to check for potentially omitted codes.

In relation to rigor and trustworthiness of the analysis, regular qualitative research team meetings were held to discuss ongoing analysis process and refine the emerging analysis structure. The research team comprised professionals from a range of backgrounds, such as nursing, clinical and health psychology, psychiatry, mental health research, and individuals with lived experiences of mental health problems and suicidality.

## 3. Results

Twenty healthcare professionals and 18 service users took part in this study. Their characteristics are presented in Tables 1, 2.

**TABLE 1:** Health professionals demographic information (*n* = 20).

Demographic	
Age (years)	Mean = 43.21 (*n* = 14), range = 33–58
Gender (female *n*, %)	12 (60)
Ethnicity (*n*, %)	White/Caucasian = 3 (15) Mixed race = 1 (5) Asian/British = 2 (10) Sudanese = 1 (5) Data not provided = 13 (65)
Profession (*n*, %)	Psychiatrist = 5 (25) Clinical psychologist = 4 (20) Commissioner = 3 (15) Third sector worker = 3 (15) Mental health nurse = 2 (10) Social worker = 2 (10) Service manager = 1 (5)
Years in current role	Mean = 7.16 (*n* = 15), Range <6 months to 12 years
Highest professional qualification (*n*, %)	Doctorate in clinical psychology = 4 (20) Master of science/masters of art = 4 (20) Registered general nurse = 3 (15) Bachelors of medicine/surgery = 3 (15) Member of royal college of psychiatry = 3 (15) Other = 3 (15)
Year qualified (*n*, %)	1980–1990 = 5 (25) 1990–2000 = 4 (20) 2000–2010 = 4 (20) 2010–2020 = 2 (10) Data not provided = 4 (20)

**TABLE 2:** Service user demographic and clinical information (*n* = 18).

Demographic	
Age (years)	Mean = 36.83, range = 18–60
Gender (female *n*, %)	10 (56)
Ethnicity (*n*, %)	White/Caucasian = 15 (83) Black African 1 (6) Asian/Jamaican = 1 (6) Mixed race, unspecified = 1 (6)
Living situation	Alone = 8 (44) With parents = 4 (22) Spouse/partner = 2 (11) Other relatives = 1 (6) Other = 3 (17)
Suicide attempts, plans and thoughts (past 6 months)	Suicide attempts, mean = 0.5, range 0–3 Suicide plans, mean = 13.44, range 0–64 Suicidal thoughts, mean = 8.22, range 1–39
Current substance use (past 3 months, *n*, %)	11 (61)

There were four key aspects of suicide-focused psychological therapy for people experiencing psychosis that healthcare professionals and service users viewed as paramount for its effective implementation, including: (i) Creating safe spaces to be understood; (ii) Gaining a voice; (iii) Accessing therapy at the right time; and (iv) Ensuring a straightforward pathway to accessing therapy (see Table 3 for themes and sub-themes).

**TABLE 3:** Structure of the themes and sub-themes.

Themes	Sub-themes
Creating safe spaces to be understood	
Gaining a voice	
Accessing therapy at the right time	
Ensuring a straightforward pathway to accessing therapy	Navigating complex services Navigating complex life circumstances

### 3.1. Creating safe spaces to be understood

From health professionals’ perspective, safety meant ensuring that clients’ safety was always maintained: “*It’d* (therapy) *definitely be welcomed in our area where* (…) *you know, huge (laughs) priority to keep people alive, to reduce distress*…” (PROF02), and the professional feeling supported by the service when working with people who feel suicidal. From service users’ perspectives, safety related to the intervention location and working with a non-judgmental therapist. For some service users, talking to someone unrelated to them who seemed non-judgmental was an integral part of therapy: *“Neutral, away from my family. You can say what you want, can’t you? And you don’t care what they think then, do you?”* (SU15).

Service users valued a private, ‘emotionally uncluttered’ environment for therapy sessions. For some, this was away from a clinical setting which was often described as overwhelming:

*“If you’re going into a medical center, I guess it’s, you know, you look at all the possible angles and all the different doors, don’t you? Like, ooh, someone’s coming at me here”* (SU17).

Travelling to attend therapy in services was particularly challenging and anxiety-provoking for some participants:

*“When I were going to therapy,* (…) *I’d be on a bus ride with all the school kids and I’d just talked about being abused at 7-year-old, and you’re sat on this bus and all these images are going on in your head. And you’ve got to get your way home. And when you already have social anxiety because there’s too much noise and stuff, I mean that’s a big struggle.”* (SU14).

Conversely, one participant preferred to attend a clinic because talking about difficult experiences made their home environment feel unclean: *“So that I don’t build up all the trauma in that flat and I’ve got to deal with all the triggers coming up in the flat again.”* (SU10). However, obtaining privacy at home was sometimes challenging and not the preferred environment for therapy for all: *“But there’s so many times where I think, well I wouldn’t be able to have you* (a therapist) *in my home because I haven’t got the privacy”* (SU14).

Service users felt that suicidal experiences can be difficult to talk about. This was due to being judged by family members: *“and my mum butts in: ‘Oh well it was one of her stupid self-harm attempts, she put her head through a window.”’* (SU10) or fear of being admitted to hospital involuntarily: *“I wouldn’t tell anybody. ‘Cause, I’m thinking, they’re going to send me away, and I didn’t want to go away”* (SU15). Therefore, to promote a feeling of safety, therapy sessions need to be delivered in an environment where service users’ experiences are not judged or dismissed.

Some service users preferred to avoid talking about suicide and described feelings of shame and embarrassment associated with suicidal thoughts and feelings: *“It’s something embarrassing, isn’t it?”* (SU03). Some service users explained that they would not talk about their suicidal experiences until they absolutely had to (e.g., in an emergency when health professionals could no longer ignore them): *“a lot of people don’t talk about their suicidal thoughts until they make an attempt, and they end up in a hospital, and they’re required to see a counselor”* (SU14). This suggests an underlying stigma both internal (i.e., self-stigma) and external, causing some people to feel shame about talking about their suicidal experiences. This may be exacerbated by response from mental health professionals, as one professional observed:

*“Lots of them (professionals) would say that they’re (service users) very selfish, that are timewasters, “he’s just going to do it, if they’re going to do it”* (…) *really lack any understanding of the situations people find themselves in”* (PROF02).

From healthcare professionals’ perspective, suicidal experiences were challenging to talk about due to perceived inability to effectively help someone with these experiences. Staff also felt unsafe working with the challenges associated with suicide due to their lack of training and fear of making people feel worse:

*“confidence and feeling that they’re (staff) not trained to do that and I think they’re told as well to some degree that they’re not allowed to do that* (…) *that they’ll make people feel worse, that you know, this general layperson view that talking about things makes things worse, so don’t talk about it and brush it under the carpet, seal over, you know, let’s not go there”* (PROF02).

On the contrary, one professional did not find it difficult to talk about suicide with service users as felt they had the experience to do so: *“I’ve been doing it for 30 years or thereabouts so er, no, I don’t find it difficult at all”* (PROF05). Service users felt that healthcare professionals avoided talking to them about suicide due to fear that it would worsen their suicidal thoughts: *“they* (professionals) *might be scared you’re going to do it*” (SU15). This alludes to a possible culture of blame within the healthcare professional community who fear the consequences of a service user telling them that they feel suicidal:

*“fears of completed suicide, and them (professionals) being criticized for not acting as they should be, so that would sort of need to come from policy really, so that they felt that they were backed up”* (PROF02).

This perception is understandable, considering the unclear pathways for staff accessing support and that it is available only in extreme situations when a suicide death occurs:

*“I think support is supposed to be offered to staff. I’ve never been in a situation where I’ve been offered it, ‘cause I’ve not actually been on duty when there’s actually been a suicide”* (PROF05).

From both service users’ and health professionals’ perspectives, having services organized and delivered in ways that helped them feel safe and understood was paramount for the successful implementation of a suicide-focused talking therapy in mental health services.

### 3.2. Gaining a voice

In their experiences of mental health services, service users spoke about feeling that they had little to no input in decisions about their care. On the other hand, healthcare professionals expressed their efforts in attempting to empower service users: *“I am also trying to empower this person to move on in their recovery, and not make people sicker. It’s a fine balance, really”* (PROF03). One professional observed that service users were feeling more empowered than ever before:

*“I think more, but I’m not saying it’s all, but I think.* (…) *it does feel like the voice of sort of service users and carers has got more power than it ever did have”* (PROF20).

Furthermore, experiences of psychosis can feel unpredictable and uncertain which can often make people feel powerless. A therapy tailored to individuals’ needs was seen to provide a sense of autonomy both over their own care, and importantly, over their mental health: “*Well, I’d just feel like it’s been done on my terms*” (SU09).

*“So, instead of just having your mental health runaway with everything and cause all the stress, the anxiety, the panic and worry that you go through, it’s about learning to regain control of your life and taking a step back and saying, “Well, okay, yes, something has happened but, yes, it’s not going to affect me, and, yes, I can get over that”” (SU10)*.

Service users reported frequently being ignored or talked over during healthcare appointments which made them feel dehumanized and disempowered: *“They* (professionals) *were telling me how I was feeling, and it annoyed me because that should be my job telling them how I feel”* (SU13). Working with health professionals who would listen to service users’ views was paramount:

*“Just to listen to my point of view, and not belittle it, and not criticize it in any way, just to listen to what I’ve got to say, and they don’t have to necessarily say anything back to me”* (SU03).

### 3.3. Accessing therapy at the right time

Healthcare professionals and service users understood the value of a talking therapy for suicidality. As one health professional noted: *“I can see multiple, you know, pathways where it* (therapy) *would really add value”* (PROF01). For service users, a suicide-focused therapy could have made a difference to the trajectory of their lives many years ago: *“now if you’d have put that in my doctors 20 years ago, my life would have gone a lot differently to what it has.”* (SU14) by improving their wellbeing and enabling them to manage their psychosis and suicidal experiences: *“Change my life, hopefully. Change my outlook. The way I see society and the way I see people.* (…) *I don’t see them in the same way as other people”* (SU09). For healthcare professionals, effectiveness also related to helping people recover to reduce the burden on mental health teams.

For some service users, therapy was viewed as being most valuable in times of crisis, as a community-based alternative to hospitalization. In this sense, therapy seems to be optimal when offered at the right time for each individual:

*“Nip it in the bud, right.* (…) *if someone is saying, ‘’I feel suicidal’ and that, get in there, you know. Don’t put them in hospital ‘cause that’s the last place that they should be in. Just give them that support, even if it’s just a call every day for 5 min, ‘How are you feeling today? Have you tried this?’ Or ‘How’s it going?’ Just find out more how to help them”* (SU13).

In contrast, healthcare professionals expressed different beliefs about the timing of therapy for service users experiencing crisis. Therapy provision was seen as challenging and possibly unhelpful to service users experiencing crisis, as one health professional noted:

*“With treatment and with her (service user’s) level of conviction reducing, that allowed for a psychological intervention to say, ‘Well,* (…) *maybe we can start challenging these things a bit,’ and that’s been very, very helpful for her. So, she’s actually improved an awful lot. But if we’d offered her psychological therapies the day she turned up in our service, I think it would’ve been not particularly effective, so it’s getting the timing right”* (PROF18).

A substantial barrier to accessing therapy from both health professionals’ and service users’ perspective was the long waiting times (e.g., many months) before therapy could begin: *“the waiting list for therapy in the community is ridiculous*” (SU10).

*“The problem that I* (…) *encounter on a regular basis from people who come to A*&*E* (Accident and Emergency) *in crisis is that they’re on a waiting list. Erm, they may have had an initial, maybe phone assessment* (…) *but then they’ve been told, ‘You’re going to have to wait 8 weeks, you’re going to have to wait a year, you’re going to have to wait 6 months,’ and if they’re struggling and in crisis, the promise of help 6 months down the line is not what they need”* (PROF05).

As such, service users felt it was futile to request therapy and healthcare professionals expressed a similar despondency about referring people for therapy. However, this could pose a problem from a commissioning perspective where demand indicates need. Without referrals or requests for psychological therapy, there appears to be no demand and, as such, no requirement to commission a new therapy.

The current system for psychological therapy for most NHS services involves a missed appointment policy whereby if a service user misses a number of appointments, they may be discharged from the service. Service users may be leading tumultuous lives due to their psychosis experiences and as such may find it challenging to attend regular appointments:

*“I think engagement. Erm, quite often if people are unwell and suicidal, they–, obviously, if they’re depressed as well, erm it might be hard getting out the house. It might be hard being organized enough to attend appointments somewhere”* (PROF05).

For many service users, this leads to a cycle of being passed between teams or from their GP back to a mental health team: *“Yeah, I go to the GP, and he’ll say, ‘But you’re under the mental health. Go and see them.’ So, then you’re like that”* (SU15).

From a commissioning or financial perspective, healthcare professionals discussed the need for therapy to come at a time when it aligns with the service’s policies, agendas, and politics. Some healthcare professionals expressed a preference for a lower intensity version of the therapy that could be provided by a care coordinator or an IAPT (Improving Access to Psychological Therapies) professional. The basis for this argument was mostly financial:

*“Quality sometimes may cost, but you’re far more likely to get funding for something that not only does it improve quality and deliver better outcomes, but you can demonstrate that it will make some kind of saving to the system”* (PROF01).

The costs associated with employing clinical psychologists or therapists to do such a therapy believed to be high but if benefits could be gained from an alternative practitioner on a lower pay grade or already working within the service, then that was seen as a potential preferred option:

*“I’m also one of the real advocates of saying you don’t all have to be highly trained therapists to deliver psychological therapies, so actually have done quite a lot of work around skill mix* (…*), and we’ve got some fabulous, fabulous outcomes”* (PROF01).

### 3.4. Ensuring a straightforward pathway to accessing therapy

Service users and healthcare professionals described navigating the pathway to accessing psychological therapies in mental health services as complex as they encountered barriers both at a service level (e.g., long therapy waiting lists) and at an individual level (e.g., managing symptoms and complex personal circumstances). These barriers made it difficult for service users to access the support they needed, and many were unaware of the help available to them.

#### 3.4.1. Navigating complex services

Service users were often not informed about the different health professional roles and staff involved in their care: *“I can’t tell you now off the top of my head whether it was a psychologist, psychiatrists”* (SU14). The lack of clarity on who to seek help from was felt to be overwhelming at times. The sense of complexity and confusion was mirrored in the professional sample. Clinical staff experienced constant changes in service delivery and implementation. This ever-changing system resulted in demoralization among staff: *“Lack of motivation, lack of clinical leadership, lack of real willingness to change.”* (PROF01). Therapy access was complicated due to issues relating to continuity of care and rigidity of psychological service provision experienced by some healthcare professionals: *“If somebody was having a particularly bad day, ‘cause we know psychology tends to be quite boundaried, would there be flexibility about appointments?”* (PROF05).

Local NHS crisis teams are available to offer immediate support for people in crisis of which participants were aware. However, most reported problems in accessing and using them: *“I don’t know why they’re called a crisis team because the number of times I’ve called them up and they can’t do anything about it*…” (SU13). Crisis teams were often perceived to struggle with low staffing levels, no financial support and low staff morale which resulted in a service that failed service users when they most require support: *“I felt as though I was getting a raw deal off the NHS ‘cause they’d just done a load of cutbacks and I’d lost the support worker that I have every day”* (SU03). One service user even set up their own support group due to lack of local crisis support: “There’s nothing local. And that’s why I ended up setting one *(support group)* up” (SU14).

General Practitioners (GPs) were viewed as a consistent point of contact compared to other resources because they sometimes provided service users with a diagnosis which facilitated access to mental health services: *“I went to see my doctor. And he diagnosed, er, schizophrenia”* (SU09). Furthermore, GPs were perceived as having more knowledge about available services and, therefore, presented a feasible gateway to psychological therapies: *“GPs refer you to the mental health service anyway.* (…) *I could go to my GP and say, ‘I want that therapy.’ ‘Oh, I’ll put your name down for it”’* (SU15). Mental health professionals, instead, viewed GPs as a gateway to medication but not necessarily talking therapies: *“I know that GPs would prescribe antidepressants in the absence of psychological therapies”* (PROF01). From healthcare professionals’ perspective, this approach was perhaps not the most optimal in addressing service users’ needs and helping them manage suicidal experiences:

*“You know the way it goes, medicate, medicate, medicate, ‘oh, look, the medication’s worked,’ where really time would have done exactly the same*…) *time and talking would have achieved exactly that”* (PROF04).

In terms of implementing a new psychological therapy, having dedicated staff who endorsed the effectiveness and success of the therapy was viewed as crucial: “*often implementation, let’s be honest, is about champions and, you know, people with passion”* (PROF01).

#### 3.4.2. Navigating complex personal circumstances

Service users experienced their mental health as complex and finding appropriate services that had the requisite expertise with respect to, for example, a long history of hearing voices was important: *“All my life*… (…) *first started hearing voices when I was 8 years old.”* (SU03), substance use: “*Yeah, they* (the voices) *were still there, but the drugs dampened them down to a sort of a level where I could function properly.”* (SU03), hospitalizations and numerous suicide attempts was challenging.

Supportive resources were available from third sector organizations which service users were aware of: *“I’ve heard about them”* (SU02). Health professionals seemed to value the importance of third sector organizations in providing support within overwhelmed services:

*“We’ve got* (…) *a very thriving third sector,* (…) *because of the huge problems we have of, of people turning up in our acute hospitals who’ve self-harmed and we commission–, so we have (organization) working in a number of our acute trusts, and we’ve seen some fantastic results”* (PROF01).

However, some service users felt that their mental health problems were so complexly intertwined that support from this sector was not always possible:

*“A lot of the charity services won’t deal with people with severe mental health because it’s too triggering for them, and they don’t have the skills or the ability to deal with severe mental health issues. So, if you’re a bit depressed because someone passed away, they can deal with it, but if you’re a bit depressed because someone passed away and you’ve got other personality disorder and psychosis in the background, they struggle to deal with it”* (SU10).

Healthcare professionals also experienced this issue where they struggled to identify how to effectively assess the severity of service users’ experiences and support them effectively when presenting with many different needs, including as an example, substance use:

*“But it’s about that judgment and perception, and which is why it’s an even heightened risk (of a suicide attempt) when you’ve got substance misuse with it”* (PROF01).

These mental health experiences require a complex package of psychological care in order to effectively meet the needs of service users. One issue to note is that of risk aversion amongst clinical staff which was not just based on clinical decision making but influenced by multiple factors often out of the control of staff working with the service user. For instance, staff described frustrating experiences where the decision whether to admit a service user to hospital following an Accident and Emergency visit related to a suicide attempt was taken out of their hands due to unavailability of hospital beds: *“one of my patients at the moment, she is hearing voices, she is actively suicidal, she’s taken an overdose recently* (…) *but we’ve no beds*…” (PROF15).

To further complicate the issue, differing views about the role of the medical model of mental health problems influenced healthcare professional and service user attitudes toward psychological therapy in general:

*“How do you build a psychological input into a crisis management, which at the moment I don’t think the psychological approach is that strong in that I think* (…) *it’s a very medical model”* (PROF01).

Overall, both service users and health care professionals recognized the usefulness and benefits of suicide-focused therapies and the need for joint efforts toward minimizing the barriers to implementing such therapies in services.

## 4. Discussion

This study addresses a gap in the literature relating to the implementation of a suicide-focused psychological talking therapy in mental health services. The results corroborate previous research on the values and usefulness of suicide-focused talking therapies (3, 17–22). Importantly, this study adds to the knowledge base by examining both healthcare professionals’ and service users’ views of the barriers and facilitators in the implementation of a suicide-focused therapy in services (see Table 4).

**TABLE 4:** Proposed barriers and facilitators to suicide-focused therapy implementation.

Implementation barrier	Implementation facilitator
Unsafe therapeutic environment	An environment where the client feels safe, listened to, and understood
Unsafe client-therapist relationship	A non-judgmental therapist who can provide space for the client to openly talk about experiences of suicide
Unsupportive healthcare staff	Kind, supportive, and non-judgmental healthcare staff
Rigid psychological service provision (i.e., inflexible appointments)	Flexible therapy appointments that consider client’s life circumstances
Unclear pathways to seeking and obtaining support in crisis	Clear signposting and information provision for available suicide-specific support for clients
Inadequate staff training and support for working with clients who feel suicidal	Provide formal training, supervision, and support for staff to provide effective care for people who feel suicidal
Service user loss of a sense of autonomy and agency	Empower and support clients in decision-making and care choices
Timing of therapy provision for service users	Collaborate with clients in decisions about the timing of their therapy (e.g., start, end, length, and location of sessions)
Timing of therapy implementation for services	Ensure the therapy is in line with the service’s goals and objectives

A key finding was that all stakeholders viewed a suicide-focused therapy as valuable for people experiencing psychosis. For this type of therapy to be successfully implemented in services, both healthcare professionals and service users agreed that three key aspects would need to be considered. The first was the provision of a therapy that fosters a sense of safety for clients to be able to openly discuss experiences of suicide with a non-judgmental and supportive therapist. Establishing services that maintain clients’ safety and wellbeing at all times was paramount for the provision of effective care (23). Similarly, in order to provide effective and safe care, staff who talk to people about suicidal experiences felt that they needed training and support from professionals in the field of suicide. This point corroborates the findings of a study examining ward staff’s views of a suicide-focused psychological therapy for psychiatric inpatients (24). The study found that formal training for staff in working effectively with people who feel suicidal would alleviate uncertainties (24). It appears that suicide-specific training and support are needed not only for staff working in mental health inpatient settings but also in community services.

Second, therapy should respect and promote service users’ sense of autonomy and empowerment in decisions about their therapy. Often service users in this study felt misunderstood, not listened to, and even ignored by professionals in decisions about their care or when they needed to talk about their experiences of suicidality. There is a breadth of research highlighting the importance and benefits of service user involvement in all aspects of care planning, including increased satisfaction and engagement (25–27). Although involving service users in decisions about their care is an established ethical and mental health policy imperative, service users continue to feel marginalized or excluded from these decisions (25, 28, 29).

Third, therapy should be offered at the right time for the individual, taking into consideration their needs and circumstances. Importantly, stakeholders’ views diverged on this topic. On the one hand, service users expressed that they would like to have access to therapy when they need it the most (e.g., in times of crisis). In contrast, some professionals felt that therapy could be ineffective during crisis or when service users were experiencing high levels of paranoia. However, provision of timely and tailored psychological interventions is imperative for people experiencing a mental health crisis (30, 31). This emphasizes the need for open discussions and mutual agreement between services and clients regarding their care and that no assumptions should be made about whether, when and where therapy should take place.

A notable finding was that both healthcare professionals and service user participants expressed concerns about the difficulty and potential consequences of talking about suicide, albeit for different reasons. For example, service users feared that they would experience stigma, be involuntarily hospitalized and/or feel worse as a result of remembering negative memories of suicidal experiences which would adversely impact their mood and mental wellbeing. This type of concern has been reported in previous research (20, 24, 32). However, talking about suicidal experiences did not have an enduring, adverse impact on participants’ lives. Indeed, research has consistently reported that talking about suicide does not induce suicidal ideation, has long-lasting positive effects and any short-term negative effects on mood do not persist (32–34). A recent study of people’s experiences of taking part in research about suicide found that participation could sometimes result in lower mood (34). However, this dip in mood was anticipated, relatively short-lived, and was outweighed by the long-lasting positive effects of participation, such as altruism (e.g., helping research and others with similar experiences) and catharsis [e.g., feeling relief after talking about suicidal experiences; (34)]. Furthermore, the Samaritans (35, 36) have championed the value of talking about suicidal experiences in their recent reports. The essence of the reports is closely linked to the findings of this study, highlighting the significance of providing a safe space and talking to people who are non-judgmental, supportive, and understanding of peoples’ difficulties. What our work adds is the value that stakeholders place on staff training and flexible services that can provide timely suicide-focused interventions for people who experience severe mental health problems, such as psychosis.

Previous research has found that healthcare professionals fear talking about suicide with service users, (24, 37) and this was observed in the current study. Like service users, the healthcare professionals in this study were concerned that talking about suicide would make people feel worse. Professionals feared that talking with service users about suicide would result in blame should a suicide death occur, or that talking about suicide is not their responsibility (24, 37). These views are exacerbated by the perceived lack of appropriate support for staff who engage in suicide talk with service users as well as the repercussions from a potential death by suicide of a service user (37, 38). The emotional impact of a client death by suicide on staff and services is profound (37, 39–41). It is compounded by the minimal support offered to staff coping with grief in the aftermath of a client suicide death (37). These are substantial barriers in the implementation of psychological interventions for suicidal experiences. Increasing provision of training from experts by experience may help change professionals’ perceptions about talking for people who feel suicidal. To create supportive workplace cultures, however, also requires organizational input and improved communication across services.

A unique aspect of this study was the involvement of individuals who experience complex mental health problems, specifically psychosis, which can make seeking and obtaining support from services a particularly challenging and distressing endeavor. The service user participants in this study described the profound impact of distressing psychosis experiences (e.g., hearing voices) on their lives, such as leaving the house, establishing relationships, or engaging in social interactions. This is an important factor in promoting and implementing suicide-focused talking therapies in services. Considering participants’ views regarding the need for such therapies, and the underfunding of services which are continuously failing to provide appropriate support for people who feel suicidal (42), the implementation of suicide-focused therapies into mainstream NHS services for people with psychosis appears imperative. A pertinent view of healthcare professionals was that any new intervention needs staff who believe in its efficacy and can champion it, in order for it to be successfully implemented in services.

### 4.1. Strengths and limitations

This study has three strengths. First, it spanned a wide geographical area and recruited participants from four NHS mental health Trusts, third sector and commissioning services, representing a diverse perspective from stakeholders. Second, and in addition to the first point, the interview topic guide for service user participants was developed and trialed in collaboration with experts by experience who advised on specific questions and interview approaches. Third, the data analysis incorporated input from academics, clinicians, and experts by experience. These approaches contributed to the trustworthiness of the analysis.

There are three limitations. First, service user participants were taking part in a randomized control trial evaluating the effectiveness of a CBT-informed suicide talking therapy. As such, the views represented those who were willing to take part in a therapy trial. Whilst it is an advantage that they had a concrete (rather than abstract) experience to discuss, nevertheless they might have had positive views of the implementation of a new therapy. Despite this, participants were able to identify potential barriers, and the views of health professionals (who were not necessarily involved in the trial) corroborated and developed these ideas. Second, most of the service user participants identified as White/Caucasian and so the findings of this study may not be generalizable to other ethnic groups, who often underserved by existing talking therapies and experience additional barriers (43, 44), and may be able to identify additional implementation challenges. Third, there were not enough data to examine the perspectives of distinct healthcare professional groups (e.g., nurses, care coordinators, and therapists), and this study can only capture a broad representation of the views of different healthcare professionals. Further research of particular communities of stakeholders is needed to understand any additional implementation barriers they may face.

## Data availability statement

The original contributions presented in this study are included in the article/supplementary material, further inquiries can be directed to the corresponding author.

## Ethics statement

The studies involving human participants were reviewed and approved by the Northwest–Greater Manchester Research Ethics Committee (17/NW/0089). The patients/participants provided their written informed consent to participate in this study.

## Author contributions

SP, YA, PG, and GH designed the study with input from grant holders on the CARMS trial [see Gooding et al. (13)]. LC, YA, and KH collected the data. SP led the analysis. LC, KH, YA, and SP drafted the manuscript. All authors discussed the analysis and writing of the manuscript, read, and approved the final manuscript.
